# Ninety days post-hospitalization evaluation of residual COVID-19 symptoms through a phone call check list

**DOI:** 10.11604/pamj.2020.37.289.27110

**Published:** 2020-12-01

**Authors:** Miguel Suárez-Robles, María del Rosario Iguaran-Bermúdez, José Luis García-Klepizg, Noel Lorenzo-Villalba, Manuel Méndez-Bailón

**Affiliations:** 1 Internal Medicine Department, San Carlos University Hospital, Madrid, Spain,; 2 Service de Médecine Interne, Diabète et Maladies Métaboliques, Hôpitaux Universitaires de Strasbourg, Strasbourg, France

**Keywords:** COVID-19, symptoms, discharge

## Abstract

An observational and descriptive study including patients discharged for COVID-19 was carried out by the COVID-19 Working Group of the Hospital Clínico San Carlos (HCSC). We aimed to identify the main symptoms after 90 days of hospital discharged. A structured interview was conducted, through a “checklist” that included symptoms within the 90 days post-discharge. A total of 134 patients were enrolled. The most frequently referred symptoms were asthenia, dyspnea and weight loss. Anxiety was the most frequent psychological symptom found through the GAD-7 scale.

## Brief

The coronavirus disease 2019 (COVID-19) has triggered a health crisis that has impacted the need for a very high number of hospitalizations in the internal medicine services of our country [1]. Spain, with more than 305,767 confirmed cases and 28,499 deaths as of July 2020, is one of the most affected countries worldwide [2]. Most of the patients hospitalized for COVID-19 in the Spanish internal medicine department have presented symptoms of fever, cough, and dyspnea. However, little is known about the evolution of these symptoms after hospital discharge in our setting [1]. For this reason, we decided to carry out this research, which aims to carry out a descriptive study of the evolution of associated symptoms after admission for COVID-19 infection.

In order to identify which of the main symptoms that patients discharged for COVID-19 present, an observational and descriptive study has been carried out by the COVID-19 working group of the Hospital Clínico San Carlos (HCSC) (Madrid). Through a telephone survey, with consecutive non-probabilistic sampling, of patients discharged for COVID-19 from this hospital, in the period between March 1^st^ and 27^th^, 2020. All patients had a positive real time reverse transcriptase polymerase chain reaction (RT-PCR) during admission for COVID-19 in our center. Subjects who did not consent to participate were excluded from the study. During the telephone survey, which was carried out during the month of June 2020, a structured interview was conducted, through a “checklist” that included symptoms within the 90 days post-discharge. The symptoms related to COVID-19, are identified by the scale of GAD-7 anxiety and follow-up variables in primary care. Readmission and hospital mortality were evaluated three months after hospital discharge. Oral informed consent was obtained from each patient to participate in the study. A descriptive analysis was performed through SPSS 21.0. Of the 134 patients surveyed, the mean age was 58.53 ± 18.53 years of which 62 were men (46.3%) and 72 women (53.7%). Of the patients included, 2 were hospitalized in the intensive care unit (ICU) and 4 were discharged with oxygen therapy. Regarding the clinical manifestations evaluated in the 90 days after hospital discharge, the most frequently referred symptom was asthenia in 73 (54.5%), dyspnea in 54 (40.3%), and weight loss in 50 (37.3%) (Table 1). In relation to the psychological symptoms evaluated, with the GAD-7 scale, it has been observed that 43.4% of the patients reported symptoms related to anxiety (Table 2). From the point of view of outpatient follow-up, 89 (66.4%) respondents reported having had outpatient follow-up by their primary care physician and in 66 (49.3%) a control analysis was performed after discharge. During this clinical follow-up, 7 patients (5.2%) had to be readmitted, 5 for bacterial respiratory infection (3.7%), and 2 for pulmonary thromboembolism and exacerbated COPD (1.5%). No patient died during follow-up. This preliminary study confirms that patients discharged for COVID-19 infection have residual symptoms of the disease in the first 90 days after discharge from hospital discharge.

**Table 1:** symptoms reported during the telephone interview 90 days after discharge after COVID-19 infection

Symptom	Frequency (N = 134)	Percentage (%)
Fatigue	73	54,5
Dyspnea	54	40,3
Loss of weight	50	37,7
Loss of appetite	36	26,9
Cough	36	26,1
Anosmia	35	26,1
Headaches	33	24,6
Arthritis	33	26,1
Palpitations	29	21,6
Dysgeusia	29	21,6
General malaise	25	18,7
Dysphonia	11	8,2
Sensitivity disorders	10	7,5
Sputum	7	5,2
Walking disturbances	5	3,7
Cutaneous manifestations	2	1.5

**Table 2:** psychological symptoms evaluated 90 days after hospital discharge for COVID-19 infection through the GAD-7 scale in the population studied

GAD 7 scale								
Symptom	Never (0)		<1/2 Days		>1/2 Days		Almost every day (3)	
	Frequency% (N = 134)		Frequency% (N = 134)		Frequency % (N = 134)		Frequency % (N = 134)	
Nervousness	67	50.0	34	25.4	22	3.4	11	8.2
Anxiety	58	43.3	43	32.1	20	14.9	13	9.7
Difficulty relaxing	82	61.2	26	19.4	16	11.9	10	7.5
Psychomotor inquiry	96	71.6	18	13.4	13	9.7	7	5.2
Irritability	97	72.4	20	14.9	12	9.0	5	3.7
Fear	88	65.7	31	23.1	6	4.5	3	6.7

These findings have been observed in the shorter term by other authors as well. Carfi A *et al*. have reported percentages of residual symptoms after COVID-19 infection from the point of view of asthenia and dyspnea similar to our series [3]. These main conditions, mainly dyspnea, coincide with the most frequent symptoms presented by patients during acute infection. However, others such as weight loss or asthenia are more referred to during convalescence from the disease. The presence of dyspnea, asthenia, and cough that some authors have defined as post-COVID-19 syndrome ranges from 10% to 65% according to the series [4]. In our research, as it is a sample of patients who have required hospital admission, it has been greater than 40%. The etiology of this post-COVID-19 syndrome is poorly understood. Persistent viremia, the absence of antibody production as well as a persistent inflammatory response of the patient may contribute to this [4]. In this sense, our study highlights that in more than 40% of the surveyed subjects, anxiety symptoms are observed. The preliminary and descriptive results of our observation emphasize the need for continuous and comprehensive care of patients with SARS-CoV-2 infection after hospital discharge. Structured telephone follow-up, the creation of virtual and monographic consultations in internal medicine services with adequate coordination with primary care could be measured to be implemented in the services of our specialty to improve the outpatient care of patients with COVID-19.
